# The Role of Chaperone-Mediated Autophagy in Hepatitis C Virus-Induced Pathogenesis

**DOI:** 10.3389/fcimb.2021.796664

**Published:** 2021-12-02

**Authors:** Chieko Matsui, Putu Yuliandari, Lin Deng, Takayuki Abe, Ikuo Shoji

**Affiliations:** ^1^ Division of Infectious Disease Control, Center for Infectious Diseases, Kobe University Graduate School of Medicine, Kobe, Japan; ^2^ Department of Clinical Microbiology, Faculty of Medicine, Udayana University, Bali, Indonesia

**Keywords:** hepatitis C virus, chaperone-mediated autophagy, CMA-targeting motif, LAMP-2A, HSC70, lysosome

## Abstract

Lysosome incorporate and degrade proteins in a process known as autophagy. There are three types of autophagy; macroautophagy, microautophagy, and chaperone-mediated autophagy (CMA). Although autophagy is considered a nonselective degradation process, CMA is known as a selective degradation pathway. All proteins internalized in the lysosome *via* CMA contain a pentapeptide KFERQ-motif, also known as a CMA-targeting motif, which is necessary for selectivity. CMA directly delivers a substrate protein into the lysosome lumen using the cytosolic chaperone HSC70 and the lysosomal receptor LAMP-2A for degradation. Hepatitis C virus (HCV) NS5A protein interacts with hepatocyte-nuclear factor 1α (HNF-1α) together with HSC70 and promotes the lysosomal degradation of HNF-1α *via* CMA, resulting in HCV-induced pathogenesis. HCV NS5A promotes recruitment of HSC70 to the substrate protein HNF-1α. HCV NS5A plays a crucial role in HCV-induced CMA. Further investigations of HCV NS5A-interacting proteins containing CMA-targeting motifs may help to elucidate HCV-induced pathogenesis.

## Introduction

The molecular mechanisms of autophagy were discovered by Prof. Yoshinori Ohsumi and his team *via* the identification of the autophagic-related genes (ATGs) in yeast in the early 1990s (Ohsumi, 2014). ATG genes are well-conserved among eukaryotes. The field of autophagy has been developed rapidly on the basis of these great discoveries. Lysosomes, together with other proteolytic systems, are involved in the constant turnover of intracellular constituents. Using this mechanism, cells eliminate aggregate-prone proteins and organelles, bulk cytoplasm, and infectious pathogens. Moreover, there is growing evidence of autophagy’s roles in cell death, differentiation, aging, growth control, antigen presentation, cell defense, and adaptation to hostile conditions (Cuervo, 2004; Mizushima, 2007).

Because of the numerous functions of autophagy in the cells, interference with this process could be associated with various human diseases. Many diseases, such as cancer, neurodegenerative diseases, metabolic dysfunction, liver diseases, and cardiovascular diseases, have been linked to disruptions in autophagy. (Levine and Kroemer, 2008; Yang and Klionsky, 2020). The failure of autophagic clearance is linked to the intracytoplasmic accumulation of misfolded and aggregate-prone protein in most adult-onset neurodegenerative disorders (Nixon, 2013). Autophagy is also critical in the adaptive immune response, specifically in the processing and presentation of major histocompatibility complex (MHC) class II antigens, in addition to its role in innate immunity (Levine and Deretic, 2007). Consequently, numerous intracellular pathogens hijack this pathway by evading autophagic detection, changing the autophagic route, and manipulating the autophagosomal compartment to their benefit (Ogawa et al., 2011).

**Figure 1:**
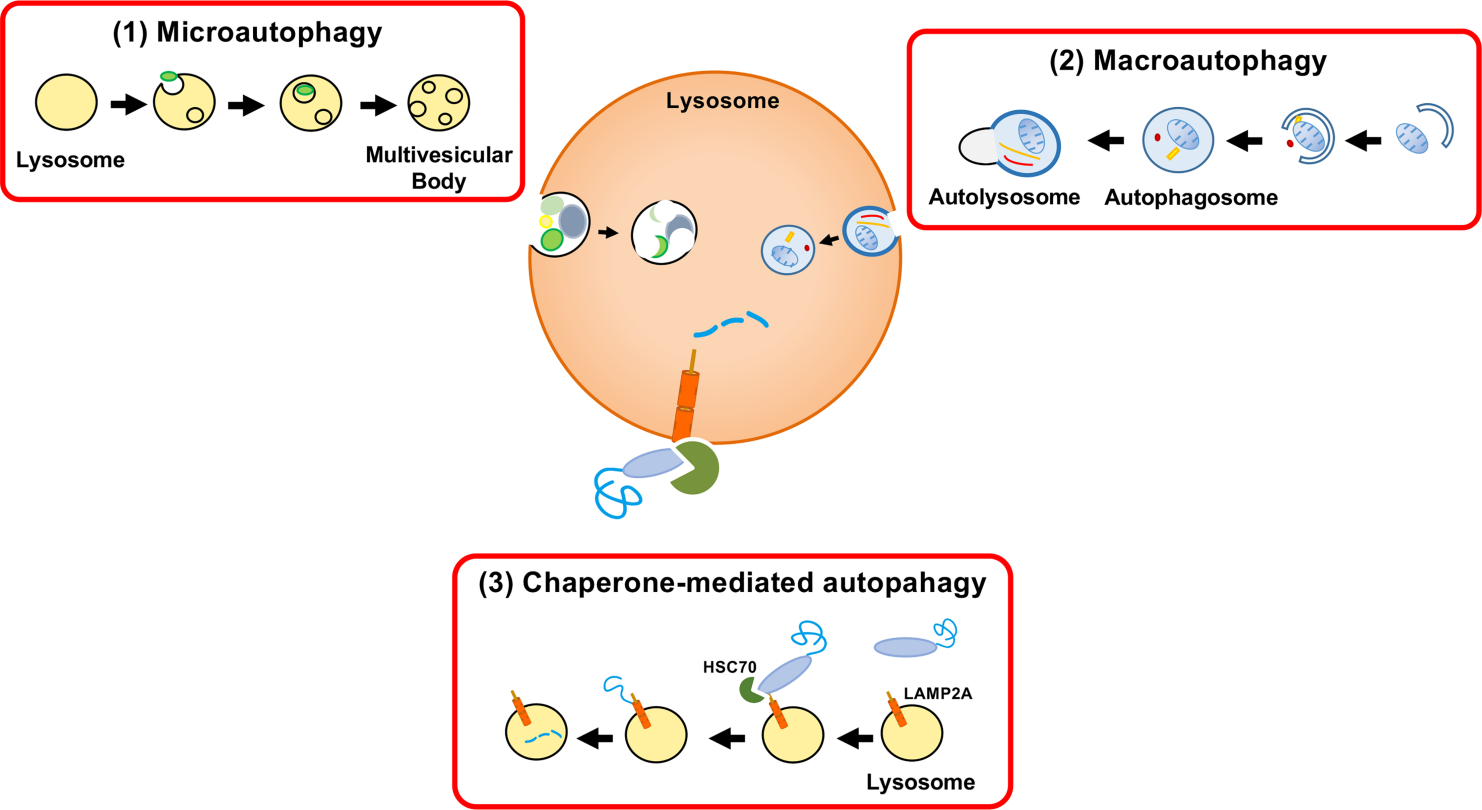
Three autophagy pathways. Proteins are incorporated into lysosomes by **(1)** macroautophagy, **(2)** microautophagy, or **(3)** chaperone-mediated autophagy (CMA). Autophagy was formerly considered a nonselective bulk degradation process. However, CMA results in the selective degradation of the cytosolic proteins. Macroautophagy involves the encapsulation of a protein with other cytosolic components and organelles in a double membrane-bound vesicle (autophagosome). The autophagosome fuses with the lysosome and the sequestered components are degraded. Microautophagy is a process in which lysosomes directly engulf cytosolic components through membrane invagination. CMA involves the selective destruction of CMA-targeting motif-containing proteins transported to lysosomes by the chaperone HSC70 and the internalization of proteins by LAMP-2A.

In mammalian cells, proteins are incorporated into lysosomes by (1) macroautophagy, (2) microautophagy, and (3) chaperone-mediated autophagy (CMA) (**Figure 1**). In macroautophagy, a protein with other cytosolic components and organelles is entrapped in a double-membrane-bound vesicle, called an autophagosome. The autophagosome fuses with the lysosome, followed by degradation of the sequestered components. In microautophagy, cytosolic components are directly ingested by lysosomes through invagination of the lysosomal membrane. The third form of autophagy, CMA, is distinct from the other types of autophagy in terms of identification of protein target by chaperone protein HSC70 and mechanism of delivery to the lysosomal lumen (Cuervo, 2004; Mizushima, 2007; Glick et al., 2010).

## The Molecular Mechanism of Chaperone-Mediated Autophagy

Although autophagy was commonly regarded in the past as a nonselective breakdown system, CMA turned out to be a type of selective autophagy. CMA selectively recognizes substrate proteins by the specific protein recognition and translocation into the lysosomal membrane in association with HSC70, a heat shock protein of around 70 kDa. All of the protein substrates degraded by CMA have a specific pentapeptide motif (KFERQ-motif; CMA-targeting motif) in their amino acid sequences (Kirchner et al., 2019; Kacal et al., 2021). A potential CMA-targeting motif can be found in 30-40% of soluble cytosolic proteins. However, additional motifs are made possible by posttranslational modifications, such as phosphorylation or acetylation, thus increasing the number of possible substrates.

**Figure 2:**
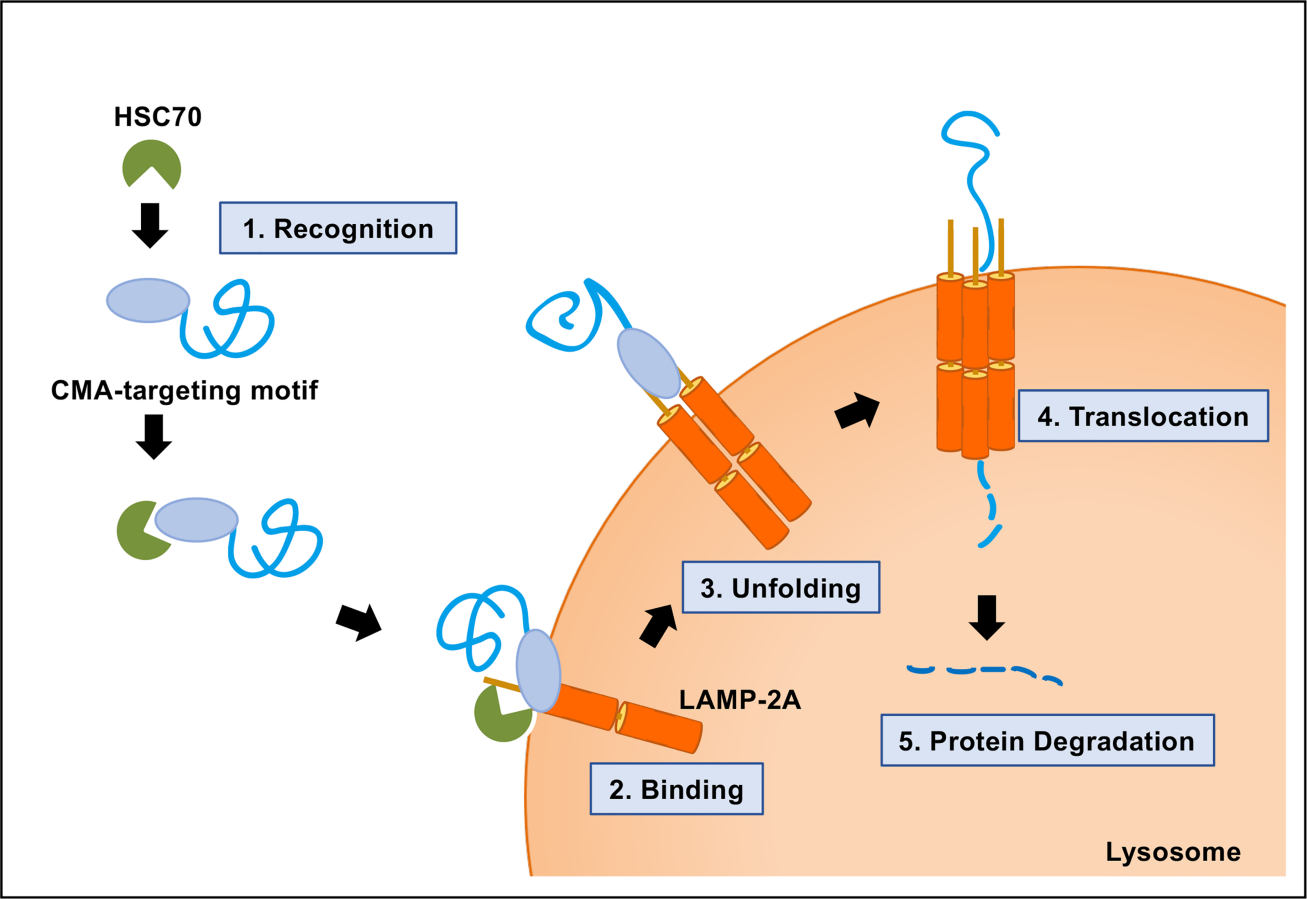
Molecular mechanism of chaperone-mediated autophagy (CMA). CMA is a five-step process. Recognition of the CMA-targeting motif in the substrate protein by HSC70 (step 1); binding of the substrate−chaperone complex to LAMP-2A (step 2); unfolding of the protein substrate (step 3); multimerization of LAMP-2A and translocation of the protein to the lysosomal lumen mediated by lysosomal HSC70 (step 4); protein degradation and disassembly of LAMP-2A multimer (step 5).

A protein-containing CMA-targeting motif is recognized by the cytosolic chaperone HSC70 (Chiang et al., 1989; Cuervo, 2011; Kirchner et al., 2019). The next step of the CMA pathway is the binding of the protein complex, a target protein and HSC70, to the cytosolic tail of lysosome-associated membrane protein type 2A (LAMP-2A) at the lysosomal membrane (Cuervo and Dice, 1996). LAMP-2A is one of the three splice variants of LAMP-2 genes: LAMP-2A, LAMP-2B, and LAMP-2C. LAMP-2A is the crucial determinant of the CMA pathway. LAMP-2A protein is necessary for CMA, but not for other types of autophagy. The production, elimination, and sub-compartmentalization of LAMP-2A receptor modulate the CMA activity in the cells (Kaushik and Cuervo, 2012; Kaushik and Cuervo, 2018; Arias and Cuervo, 2020). Multimerization of LAMP-2A is required for translocation of unfolded substrate protein into the lysosome lumen (Cuervo and Wong, 2014). Finally, the target protein is rapidly degraded in the lysosome, followed by the dissociation of the translocation complex and monomerization of LAMP-2A (**Figure 2**).

## CMA and eMI

**Figure 3:**
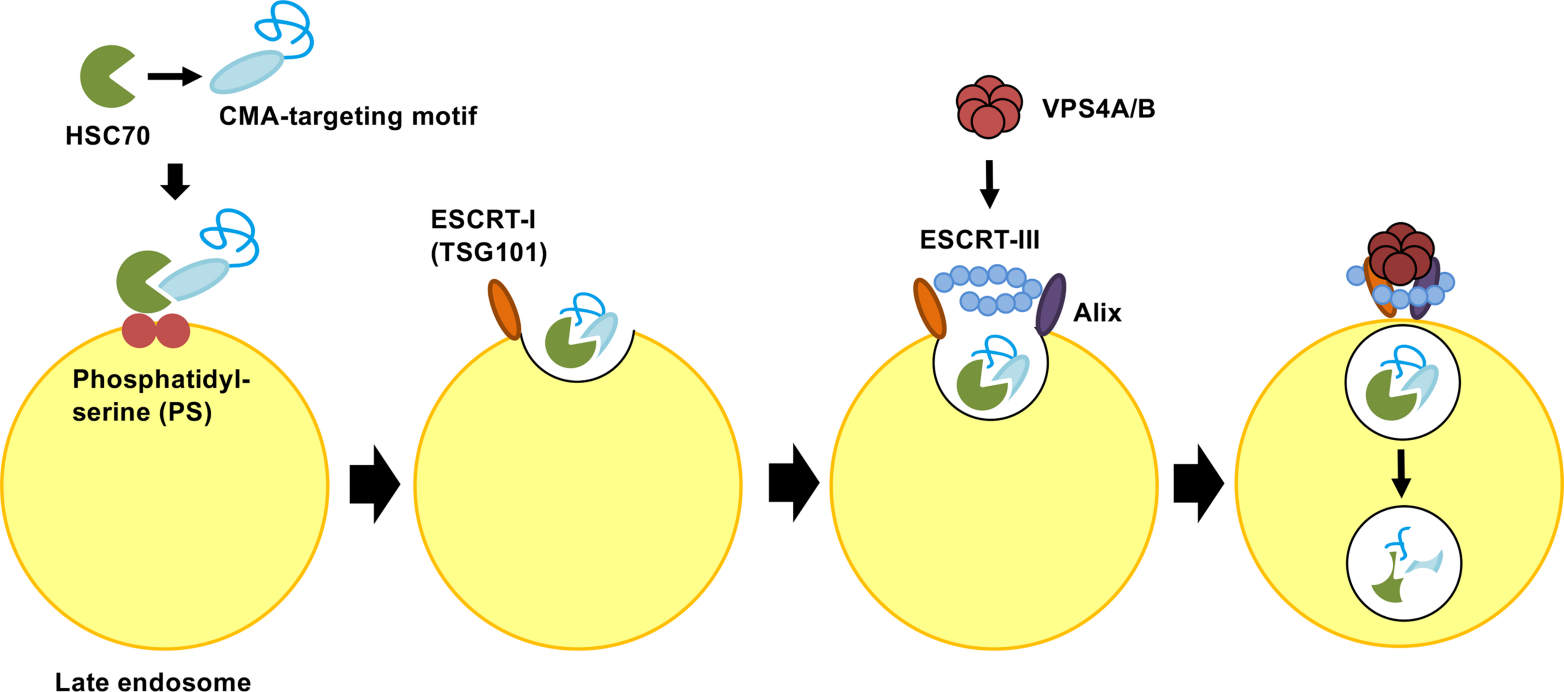
Molecular mechanism of endosomal microautophagy (eMI). Multifunctional chaperone HSC70 recognizes protein bearing CMA-targeting motifs. Upon cargo binding, HSC70 directly interacts with phosphatidylserine (PS) in the endosomal membrane. HSC70 is internalized along with protein into microvesicles *via* the coordinating functions of ESCRT I (TSG101), ESCRT III, VPS4A/B, and Alix. The degradation of microvesicles occurs in the endosomal lumen or lysosome *via* endosome−lysosome fusion. ESCRT, endosomal sorting complex required for transport.

Endosomal microautophagy (eMI) is another type of selective protein degradation using HSC70 for recognition of a CMA-targeting motif. In contrast to CMA, the unfolding and LAMP-2A binding of cytosolic proteins is not required for eMI. A substrate protein for eMI is sequestered by the formation of the invagination in the surface of the endosomal membrane through the coordinated function of ESCRT I (TSG101) and three accessory proteins: VPS4A, VPS4B and Alix. After binding to a substrate protein, HSC70 interacts with phosphatidylserine of the endosomal membrane. HSC70 is internalized along with the substrate protein in microvesicles involved in the endosomal sorting complex required for transport (ESCRT). Substrate proteins in vesicles are degraded in the late endosome (**Figure 3**). However, it’s still unclear whether the entire ESCRT machinery is necessary for the eMI pathway (Tekirdag and Cuervo, 2018; Sahu, et al., 2011). In the CMA pathway, HSC70 is released back to the cytosol after the substrate is transferred back to LAMP-2A. On the other hand, HSC70 is internalized and degraded with the target protein in the eMI pathway (Sahu, et al., 2011; Madrigal-Matute and Cuervo, 2016). Although both CMA and eMI use the CMA-targeting motif for substrate recognition, the substrates of CMA and eMI do not fully overlap. The CMA-targeting motif is necessary and sufficient for HSC70-induced degradation on CMA, whereas the CMA-targeting motif is not sufficient for the targeting degraded proteins in eMI (Tekirdag and Cuervo, 2018; Kichner et al., 2019). Microtubule-associated protein Tau, involved in axoplasmic transport in normal neurons, is known to be degraded by both eMI and CMA (Mukherjee et al., 2016). The intrinsic properties of the substrate protein may be accountable for the shifting between these two pathways. Because CMA and eMI require different receptors to transport the target protein to the appropriate location of degradation, the knock-down of each receptor will assist in the analysis of protein degradation; that is, the knock-down of LAMP2A membrane protein increases the amount of target protein in the CMA pathway. On the other hand, the substrate protein level increases in the eMI pathway after the knock-down of the VPS4A/B protein (Tekirdag and Cuervo, 2018).

## CMA and Human Diseases

Many studies have discovered the association of impairment of the CMA process and human diseases. CMA is known to be involved in Parkinson’s disease (Wong and Cuervo, 2010), Huntington’s disease (Bauer et al., 2010; Koga et al., 2011; Qi et al., 2012), Alzheimer’s disease (Liu et al., 2009; Wang et al., 2009), prostate cancer (Lv et al., 2011), and renal diseases (Sooparb et al., 2004). *Salmonella enterica*, an invasive intracellular bacterium, exploits LAMP-2A and HSC70 to promote proliferation (Singh et al., 2017). This bacterium activates the CMA pathway to degrade tripartite motif (TRIM) 21, an E3 ubiquitin ligase which is involved in regulating the IFN-I response, to escape the host immune system (Hos et al., 2020).

## CMA and Hepatitis C Virus Infection

HCV is an enveloped, positive single-stranded RNA virus that belongs to the *Flaviviridae* family, *Hepacivirus* genus (Ray et al., 2013). The HCV genome consists of a 9.6kb RNA encoding a polyprotein of 3,010 amino acids (aa). The polyprotein is cleaved into three structural proteins (core, envelope 1 [E1], and envelope 2 [E2]) and seven nonstructural proteins (p7, nonstructural protein 2 [NS2], NS3, NS4A, NS4B, NS5A, NS5B) proteins by viral proteases and host signal peptidase (Ray et al., 2013). The structural proteins are responsible for the formation of virions, whereas the nonstructural protein is involved in viral replication (Lohmann et al., 1999; Blight et al., 2000). Approximately 56 million people (0.8% of the global population) are chronically infected with HCV (World Health Organization, 2021). Within two or three decades after infection, around 20% of HCV carriers will develop cirrhosis and hepatocellular carcinoma, either of which requires liver transplantation (Roudot-Thoraval, 2021).

Several studies have associated both structural and nonstructural HCV proteins with macroautophagy (Guevin et al., 2010; Su et al., 2011; Wang et al., 2014; Lee et al., 2019). As macroautophagy serves various functions in the host cell, it also serves to sustain HCV life cycle. However, the molecular mechanism by which HCV induces macroautophagy is still unclear (Ke and Chen, 2014).

HCV infection causes not only intrahepatic diseases but also extrahepatic manifestations, such as metabolic disorders (Ramos-Casals et al., 2017; Koike, 2009). We reported that HCV infection suppresses GLUT2 gene expression *via* selective lysosomal degradation of transcription factor HNF-1α protein (Matsui et al., 2012). HCV infection induces lysosomal degradation of this protein *via* interaction with NS5A protein (Matsui et al., 2015). We then discovered the HCV-induced selective degradation of HNF-1α *via* CMA (Matsui et al., 2018).

Other groups reported that CMA targets IFNAR1 degradation in the lysosome in free fatty acids-treated HCV cell culture (Kurt et al., 2015; Dash et al., 2016). They also reported that CMA promotes Beclin1 degradation through Nrf2 signaling in persistently infected HCV cell cultures (Aydin et al., 2018; Dash et al., 2020).

It has been reported that ER stress induces CMA *via* activation of p38 MAPK, resulting in phosphorylation of LAMP-2A and accumulation of LAMP-2A on lysosomal membrane (Li et al., 2017). Dash et al. (Dash et al., 2019) have described that ER stress uses the p38 MAPK-CMA pathway to maintain cell survival under stress. HCV was reported to induce ER stress (Wang et al., 2019). Therefore, it remains to be elucidated whether HCV infection induces ER stress to activate CMA pathway.

## Molecular Mechanism of HCV-Induced CMA Pathway

**Figure 4:**
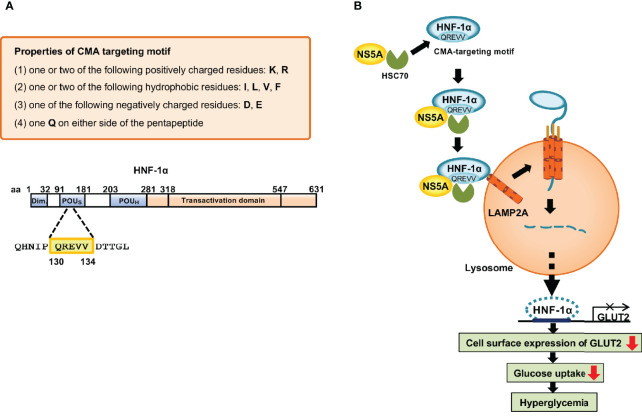
Mechanism of the HCV-induced degradation of HNF-1α *via* CMA. **(A)** The basic requirements of the CMA-targeting motif. One glutamine residue (Q) flanked on either side by one or two basic amino acids (K or R), an acidic amino acid (D or E), and one or two bulky hydrophobic amino acids (F, I, L or V). Following this rule, we identified the CMA-targeting motif (^130^QREVV^134^) in the POUs domain of HNF-1α. **(B)** HCV NS5A interacts with HSC70 and recruits HSC70 to HNF-1α protein. HSC70 binds to the CMA-targeting motif of HNF-1α. The protein complexes are delivered to the surface of the lysosomal membrane to bind to LAMP-2A. Once HNF-1α binds to LAMP2A, HNF-1α unfolds and crosses the lysosomal membrane with the assistance of LAMP-2A. Finally, HNF-1α is degraded by lysosomal proteases, resulting in the downregulation of the GLUT2 transcription. GLUT2 mRNA levels and GLUT2 expression decrease, resulting in decreased glucose uptake to the cell, which in turn leads to hyperglycemia.

To clarify the molecular mechanism underlying the HCV-induced CMA pathway, we searched for the CMA-targeting motif within HNF-1α. We identified the CMA-targeting motif of HNF-1α raging from aa 130 to 134, QREVV (**Figure 4**). HSC70 binds HNF-1α *via* its CMA-targeting motif, ^130^QREVV^134^. Protein Complex NS5A/HSC70/HNF-1α is transported to the lysosome, resulting in the association of HNF-1α with LAMP-2A. HNF-1α crosses the membrane with the assistance of LAMP-2A. HNF-1α is degraded in the lysosome. We propose that HCV-induced HNF-1α degradation *via* CMA suppresses GLUT2 gene expression, leading to the downregulation of cell surface expression of GLUT2 and the disruption of glucose uptake into the cells (Matsui et al., 2012; Matsui et al., 2015; Matsui et al., 2018) (**Figure 4**).

## Analysis of HCV-Induced CMA Pathway

### Detection of the CMA-Targeting Motif in the Sequence of Substrate Protein

The CMA-targeting motif in the amino acid sequence of the substrate protein is essential for the interaction between HSC70 and a substrate protein. Cuervo’s group proposed the basic requirements for the CMA-targeting motif (Kaushik and Cuervo, 2018; Kirchner et al., 2019). A CMA-targeting motif contains one or two of the positively charged residues: lysine (K) or arginine (R); one or two of the hydrophobic residues: phenylalanine (F), isoleucine (I), leucine (L), or valine (V); one of the negatively charged residues: aspartic acid (D) or glutamic acid (E); and one glutamine (Q) on either side of the pentapeptide (**Figure 4**). The removal of the pentapeptide amino acid in a target protein inhibited its lysosomal degradation, underscoring the importance of this motif in the CMA pathway (Dice et al., 1990; Wing et al., 1991; Kaushik and Cuervo, 2012). Free Web-based software, KFERQ finder V0.8 (https://rshine.einsteinmed.org/) was developed by Cuervo’s group to facilitate rapid identification of this motif in any protein sequences (Kirchner et al., 2019).

### HCV NS5A Plays a Crucial Role in HCV Induced-CMA Pathway

HCV infection enhances the interaction between HSC70 and HNF-1α (Matsui et al., 2018). The selective lysosomal degradation of HNF-1α protein is induced by HCV NS5A. Domain I of NS5A plays a crucial role in the interaction with HNF-1α and the degradation of HNF-1α protein (Matsui et al., 2015).

HCV NS5A interacts with HSC70 to promote the recruitment of HSC70 to the substrate protein. NS5A binds both HSC70 and the substrate protein. HSC70 binds to its substrate protein *via* the CMA-targeting motif. Protein Complex NS5A/HSC70/substrate protein is transported to the lysosomal membrane, resulting in association with LAMP-2A. The substrate protein can then cross the membrane with the assistance of LAMP-2A.

### HSC70 and LAMP-2A Are Key Components of the CMA Machinery

The cytosolic chaperone HSC70 recognizes the host protein *via* the CMA-targeting motif (Bonam et al., 2019). The HCV-induced degradation of HNF-1α is restored by the siRNA knockdown of HSC70. To investigate whether LAMP-2A plays a role in the HCV-induced degradation of HNF-1α, we knocked down LAMP-2A mRNA by siRNA. The knockdown recovered the level of substrate protein in HCV-infected cells. This result suggests that HNF-1α is degraded through CMA, but not through eMI (Matsui et al., 2018).

### HCV NS5A Is Colocalized With a Substrate Protein in the Lysosome

In HCV-uninfected cells, HNF-1α is localized mainly in the nucleus. When cells are infected with HCV, HNF-1α is localized in both the nucleus and in the cytoplasm. Since HCV NS5A is localized in the cytoplasm, NS5A is colocalized with HNF-1α protein in the cytoplasm in HCV-infected cells. HCV NS5 protein binds to HNF-1α and retains it in the cytoplasm, which may facilitate the CMA-induced degradation of HNF-1α. We performed immunofluorescence staining to confirm the subcellular colocalization of NS5A and HNF-1α in the lysosome. When cells were treated with a lysosomal enzyme inhibitor, pepstatin A, the colocalization of HNF-1α protein with HCV NS5A was detected in the lysosome.

### Treatment of Lysosomal Inhibitor

NH_4_Cl, an inhibitor of lysosomal proteolysis, is known to neutralize the acidic lysosomal pH. When HCV infection induces protein degradation of the substrate through the CMA pathway, treatment of the cells with 5mM NH_4_Cl restores the substrate protein levels.

## CMA and Other Viruses

As an obligate intracellular pathogen, viral replication depends strongly on the host machinery. Viruses utilize the autophagy system of the host, including the CMA pathway, to maintain their life cycles. In addition, some viruses interact with HSC70 and its co-chaperones, resulting in either a positive or negative life cycle regulator (Kaushik and Cuervo, 2018; Wang et al., 2020). Recently, it was reported that the NS2A protein of Zika virus promoted degradation of karyopherin subunit alpha 2 (KPNA2) *via* CMA, resulting in increased Zika virus production. The KNPA2 protein level was restored in the LAMP-2A knockdown cells infected with the Zika virus, indicating the important role of the CMA pathway in this viral replication (He et al., 2020).

## Conclusions and Future Perspectives

We clarified the molecular mechanism underlying HCV-induced CMA. We demonstrated that HCV NS5A interacts with chaperone HSC70, and recruits it to the substrate protein for lysosomal degradation *via* CMA, thereby facilitating HCV pathogenesis. There are two crucial requirements of the substrates for HCV-induced CMA; NS5A binding and a CMA-targeting motif. We and other groups have been investigating NS5A-interacting proteins (Matsui et al., 2012; Sianipar et al., 2015; Ross-Thriepland and Harris, 2015; Chen et al., 2016; Minami et al., 2017; Abe et al., 2020). Using software, we can easily do a search to determine whether the NS5A-interacting proteins contain potential CMA-targeting motifs. We have already found that at least 40 NS5A-binding proteins that contain potential CMA-targeting motifs. Further identification of novel substrates for HCV-induced CMA pathways is required to clarify the physiological relevance of the CMA-dependent degradation of host proteins in HCV infection. We provided evidence suggesting that NS5A-HSC70 complex is important for HCV-induced CMA. Small molecules that can inhibit the NS5A-HSC70 interaction may contribute to the therapeutic strategy for HCV-induced pathogenesis.

## Author Contributions

CM and PY outlined and wrote the first draft. IS edited and finalized the manuscript. CM, PY, LD, and TA conceived and produced the figures. All authors contributed to the article and approved the submitted version.

## Funding

The writing of this review was made possible by grants from the Japan Agency for Medical Research and Development (AMED) under grant number JP21fk0210090, JP21fk0210053, and JP21fk0310104, by a grant from the KAKENHI under grant number 20K07514. PY was supported by the Program for Nurture of Next Generation Leaders Guiding Medical Innovation in Asia of the Ministry of Education, Culture, Sports, Science, and Technology (MEXT) of Japan. CM was supported by the KAKENHI under grant number 19K16671. CM, LD, and TA were supported by grants from Hyogo Science and Technology Association.

## Conflict of Interest

The authors declare that the research was conducted in the absence of any commercial or financial relationships that could be construed as a potential conflict of interest.

## Publisher’s Note

All claims expressed in this article are solely those of the authors and do not necessarily represent those of their affiliated organizations, or those of the publisher, the editors and the reviewers. Any product that may be evaluated in this article, or claim that may be made by its manufacturer, is not guaranteed or endorsed by the publisher.

## References

[B1] Abe T. Minami N. Bawono R. G. Matsui C. Deng L. Fukuhara T.. (2020). ISGylation of Hepatitis C Virus NS5A Protein Promotes Viral RNA Replication via Recruitment of Cyclophilin A. J. Virol. 94, e00532–e00520. doi: 10.1128/JVI.00532-20 32727878 PMC7527057

[B2] Arias E. Cuervo A. M. (2020). Pros and Cons of Chaperone-Mediated Autophagy in Cancer Biology. Trends Endocrinol. Metab. 31, 53–66. doi: 10.1016/j.tem.2019.09.007 31699565 PMC7020649

[B3] Aydin Y. Stephens C. M. Chava S. Heidari Z. Panigrahi R. Williams D. D.. (2018). Chaperone-Mediated Autophagy Promotes Beclin1 Degradation in Persistently Infected Hepatitis C Virus Cell Culture. Am. J. Pathol. 188, 2339–2355. doi: 10.1016/j.ajpath.2018.06.022 30075149 PMC6168955

[B4] Bauer P. O. Goswami A. Wong H. K. Okuno M. Kurosawa M. Yamada M.. (2010). Harnessing Chaperone-Mediated Autophagy for the Selective Degradation of Mutant Huntingtin Protein. Nat. Biotechnol. 28, 256–263. doi: 10.1038/nbt.1608 20190739

[B5] Blight K. J. Kolykhalov A. A. Rice C. M. (2000). Efficient Initiation of HCV RNA Replication in Cell Culture. Science 290, 1972–1974. doi: 10.1126/science.290.5498.1972 11110665

[B6] Bonam S. R. Ruff M. Muller S. (2019). HSPA8/HSC70 in Immune Disorders: A Molecular Rheostat That Adjusts Chaperone-Mediated Autophagy Substrates. Cells 8, 849. doi: 10.3390/cells8080849 PMC6721745 31394830

[B7] Chen M. Gan X. Yoshino K. Kitakawa M. Shoji I. Deng L.. (2016). Hepatitis C Virus NS5A Protein Interacts With Lysine Methyltransferase SET and MYND Domain-Containing 3 and Induces Activator Protein 1 Activation. Microbiol. Immunol. 60, 407–417. doi: 10.1111/1348-0421.12383 27080060

[B8] Chiang H. L. Terlecky S. R. Plant C. P. Dice J. F. (1989). A Role for a 70-Kilodalton Heat Shock Protein in Lysosomal Degradation of Intracellular Proteins. Science 246, 382–385. doi: 10.1126/science.2799391 2799391

[B9] Cuervo A. M. (2004). Autophagy: In Sickness and in Health. Trends Cell Biol. 14, 70–77. doi: 10.1016/j.tcb.2003.12.002 15102438

[B10] Cuervo A. M. (2011). Chaperone-Mediated Autophagy: Dice’s ‘Wild’ Idea About Lysosomal Selectivity. Nat. Rev. Mol. Cell Biol. 12, 535–541. doi: 10.1038/nrm3150 21750569

[B11] Cuervo A. M. Dice J. F. (1996). A Receptor for the Selective Uptake and Degradation of Proteins by Lysosomes. Science 273, 501–503. doi: 10.1126/science.273.5274.501 8662539

[B12] Cuervo A. M. Wong E. (2014). Chaperone-Mediated Autophagy: Roles in Disease and Aging. Cell Res. 24, 92–104. doi: 10.1038/cr.2013.153 24281265 PMC3879702

[B13] Dash S. Aydin Y. Moroz K. (2019). Chaperone-Mediated Autophagy in the Liver: Good or Bad? Cells 8, 1308. doi: 10.3390/cells8111308 PMC6912708 31652893

[B14] Dash S. Aydin Y. Wu T. (2020). Integrated Stress Response in Hepatitis C Promotes Nrf2-Related Chaperone-Mediated Autophagy: A Novel Mechanism for Host-Microbe Survival and HCC Development in Liver Cirrhosis. Semin. Cell Dev. Biol. 101, 20–35. doi: 10.1016/j.semcdb.2019.07.015 31386899 PMC7007355

[B15] Dash S. Chava S. Aydin Y. Chandra P. K. Ferraris P. Chen W.. (2016). Hepatitis C Virus Infection Induces Autophagy as a Prosurvival Mechanism to Alleviate Hepatic ER-Stress Response. Viruses 8, 150. doi: 10.3390/v8050150 PMC4885105 27223299

[B16] Dice J. F. Terlecky S. R. Chiang H. L. Olson T. S. Isenman L. D. Short-Russell S. R.. (1990). A Selective Pathway for Degradation of Cytosolic Proteins by Lysosomes. Semin. Cell Biol. 1, 449–455. 2103896

[B17] Glick D. Barth S. Macleod K. F. (2010). Autophagy: Cellular and Molecular Mechanisms. J. Pathol. 221, 3–12. doi: 10.1002/path.2697 20225336 PMC2990190

[B18] Guevin C. Manna D. Belanger C. Konan K. V. Mak P. Labonte P. (2010). Autophagy Protein ATG5 Interacts Transiently With the Hepatitis C Virus RNA Polymerase (NS5B) Early During Infection. Virology 405, 1–7. doi: 10.1016/j.virol.2010.05.032 20580051 PMC2925245

[B19] He J. Yang L. Chang P. Yang S. Lin S. Tang Q.. (2020). Zika Virus NS2A Protein Induces the Degradation of KPNA2 (Karyopherin Subunit Alpha 2) *Via* Chaperone-Mediated Autophagy. Autophagy 16, 2238–2251. doi: 10.1080/15548627.2020.1823122 32924767 PMC7751636

[B20] Hos N. J. Fischer J. Hos D. Hejazi Z. Calabrese C. Ganesan R.. (2020). TRIM21 Is Targeted for Chaperone-Mediated Autophagy During Salmonella Typhimurium Infection. J. Immunol. 205, 2456–2467. doi: 10.4049/jimmunol.2000048 32948684 PMC7576115

[B21] Kacal M. Zhang B. Hao Y. Norberg E. Vakifahmetoglu-Norberg H. (2021). Quantitative Proteomic Analysis of Temporal Lysosomal Proteome and the Impact of the KFERQ-Like Motif and LAMP2A in Lysosomal Targeting. Autophagy 1–10. doi: 10.1080/15548627.2021.1876343 PMC8632328 33446043

[B22] Kaushik S. Cuervo A. M. (2012). Chaperone-Mediated Autophagy: A Unique Way to Enter the Lysosome World. Trends Cell Biol. 22, 407–417. doi: 10.1016/j.tcb.2012.05.006 22748206 PMC3408550

[B23] Kaushik S. Cuervo A. M. (2018). The Coming of Age of Chaperone-Mediated Autophagy. Nat. Rev. Mol. Cell Biol. 19, 365–381. doi: 10.1038/s41580-018-0001-6 29626215 PMC6399518

[B24] Ke P. Y. Chen S. S. (2014). Autophagy in Hepatitis C Virus-Host Interactions: Potential Roles and Therapeutic Targets for Liver-Associated Diseases. World J. Gastroenterol. 20, 5773–5793. doi: 10.3748/wjg.v20.i19.5773 24914338 PMC4024787

[B25] Kirchner P. Bourdenx M. Madrigal-Matute J. Tiano S. Diaz A. Bartholdy B. A.. (2019). Proteome-Wide Analysis of Chaperone-Mediated Autophagy Targeting Motifs. PloS Biol. 17, e3000301. doi: 10.1371/journal.pbio.3000301 31150375 PMC6561683

[B26] Koga H. Martinez-Vicente M. Arias E. Kaushik S. Sulzer D. Cuervo A. M. (2011). Constitutive Upregulation of Chaperone-Mediated Autophagy in Huntington’s Disease. J. Neurosci. 31, 18492–18505. doi: 10.1523/jneurosci.3219-11.2011 22171050 PMC3282924

[B27] Koike K. (2009). Steatosis, Liver Injury, and Hepatocarcinogenesis in Hepatitis C Viral Infection. J. Gastroenterol. 44 Suppl 19, 82–88. doi: 10.1007/s00535-008-2276-4 19148799

[B28] Kurt R. Chandra P. K. Aboulnasr F. Panigrahi R. Ferraris P. Aydin Y.. (2015). Chaperone-Mediated Autophagy Targets IFNAR1 for Lysosomal Degradation in Free Fatty Acid Treated HCV Cell Culture. PloS One 10, e0125962. doi: 10.1371/journal.pone.0125962 25961570 PMC4427131

[B29] Lee J. S. Tabata K. Twu W. I. Rahman M. S. Kim H. S. Yu J. B.. (2019). RACK1 Mediates Rewiring of Intracellular Networks Induced by Hepatitis C Virus Infection. PloS Pathog. 15, e1008021. doi: 10.1371/journal.ppat.1008021 31525236 PMC6762199

[B30] Levine B. Deretic V. (2007). Unveiling the Roles of Autophagy in Innate and Adaptive Immunity. Nat. Rev. Immunol. 7, 767–777. doi: 10.1038/nri2161 17767194 PMC7097190

[B31] Levine B. Kroemer G. (2008). Autophagy in the Pathogenesis of Disease. Cell 132, 27–42. doi: 10.1016/j.cell.2007.12.018 18191218 PMC2696814

[B32] Liu H. Wang P. Song W. Sun X. (2009). Degradation of Regulator of Calcineurin 1 (RCAN1) is Mediated by Both Chaperone-Mediated Autophagy and Ubiquitin Proteasome Pathways. FASEB J. 23, 3383–3392. doi: 10.1096/fj.09-134296 19509306

[B33] Li W. Zhu J. Dou J. She H. Tao K. Xu H.. (2017). Phosphorylation of LAMP2A by P38 MAPK Couples ER Stress to Chaperone-Mediated Autophagy. Nat. Commun. 8, 1763. doi: 10.1038/s41467-017-01609-x 29176575 PMC5701254

[B34] Lohmann V. Körner F. Koch J. Herian U. Theilmann L. Bartenschlager R. (1999). Replication of Subgenomic Hepatitis C Virus RNAs in a Hepatoma Cell Line. Science 285, 110–113. doi: 10.1126/science.285.5424.110 10390360

[B35] Lv L. Li D. Zhao D. Lin R. Chu Y. Zhang H.. (2011). Acetylation Targets the M2 Isoform of Pyruvate Kinase for Degradation Through Chaperone-Mediated Autophagy and Promotes Tumor Growth. Mol. Cell. 42, 719–730. doi: 10.1016/j.molcel.2011.04.025 21700219 PMC4879880

[B36] Madrigal-Matute J. Cuervo A. M. (2016). Regulation of Liver Metabolism by Autophagy. Gastroenterology 150, 328–339. doi: 10.1053/j.gastro.2015.09.042 26453774 PMC4728051

[B37] Matsui C. Deng L. Minami N. Bonam T. Koike K. Shoji I. (2018). Hepatitis C Virus NS5A Protein Promotes the Lysosomal Degradation of Hepatocyte Nuclear Factor 1alpha via Chaperone-Mediated Autophagy. J. Virol. 92, e00639–e00618. doi: 10.1128/JVI.00639-18 29695419 PMC6002715

[B38] Matsui C. Rosalyn Sianipar I. Minami N. Deng L. Hotta H. Shoji I. (2015). A Single-Amino-Acid Mutation in Hepatitis C Virus NS5A Disrupts Physical and Functional Interaction With the Transcription Factor HNF-1alpha. J. Gen. Virol. 96, 2200–2205. doi: 10.1099/vir.0.000179 25957097

[B39] Matsui C. Shoji I. Kaneda S. Sianipar I. R. Deng L. Hotta H. (2012). Hepatitis C Virus Infection Suppresses GLUT2 Gene Expression via Downregulation of Hepatocyte Nuclear Factor 1alpha. J. Virol. 86, 12903–12911. doi: 10.1128/JVI.01418-12 22993150 PMC3497665

[B40] Minami N. Abe T. Deng L. Matsui C. Fukuhara T. Matsuura Y.. (2017). Unconjugated Interferon-Stimulated Gene 15 Specifically Interacts With the Hepatitis C Virus NS5A Protein via Domain I. Microbiol. Immunol. 61, 287–292. doi: 10.1111/1348-0421.12493 28543875

[B41] Mizushima N. (2007). Autophagy: Process and Function. Genes Dev. 21, 2861–2873. doi: 10.1101/gad.1599207 18006683

[B42] Mukherjee A. Patel B. Koga H. Cuervo A. M. Jenny A. (2016). Selective Endosomal Microautophagy is Starvation-Inducible in Drosophila. Autophagy 12, 1984–1999. doi: 10.1080/15548627.2016.1208887 27487474 PMC5103356

[B43] Nixon R. A. (2013). The Role of Autophagy in Neurodegenerative Disease. Nat. Med. 19, 983–997. doi: 10.1038/nm.3232 23921753

[B44] Ogawa M. Mimuro H. Yoshikawa Y. Ashida H. Sasakawa C. (2011). Manipulation of Autophagy by Bacteria for Their Own Benefit. Microbiol. Immunol. 55, 459–471. doi: 10.1111/j.1348-0421.2011.00343.x 21707736

[B45] Ohsumi Y. (2014). Historical Landmarks of Autophagy Research. Cell Res. 24, 9–23. doi: 10.1038/cr.2013.169 24366340 PMC3879711

[B46] Qi L. Zhang X. D. Wu J. C. Lin F. Wang J. Difiglia M.. (2012). The Role of Chaperone-Mediated Autophagy in Huntingtin Degradation. PloS One 7, e46834. doi: 10.1371/journal.pone.0046834 23071649 PMC3469570

[B47] Ramos-Casals M. Zignego A. L. Ferri C. Brito-Zeron P. Retamozo S. Casato M.. (2017). Evidence-Based Recommendations on the Management of Extrahepatic Manifestations of Chronic Hepatitis C Virus Infection. J. Hepatol. 66, 1282–1299. doi: 10.1016/j.jhep.2017.02.010 28219772

[B48] Ray S. C. Bailey J. R. Thomas,. D. L. (2013). “Hepatitis C Virus,” in In Fields’ Virology, 6th edn. Eds. Knipe D. M. Howley P. M. (Philadelphia, PA: Wolters Kluwer Health/Lippincott Williams & Wilkins), 795–824.

[B49] Ross-Thriepland D. Harris M. (2015). Hepatitis C Virus NS5A: Enigmatic But Still Promiscuous 10 Years on! J. Gen. Virol. 96, 727–738. doi: 10.1099/jgv.0.000009 25481754

[B50] Roudot-Thoraval F. (2021). Epidemiology of Hepatitis C Virus Infection. Clin. Res. Hepatol. Gastroenterol. 45, 101596. doi: 10.1016/j.clinre.2020.101596 33610022

[B51] Sahu R. Kaushik S. Clement C. C. Cannizzo E. S. Scharf B. Follenzi A.. (2011). Microautophagy of Cytosolic Proteins by Late Endosomes. Dev. Cell. 20, 131–139. doi: 10.1016/j.devcel.2010.12.003 21238931 PMC3025279

[B52] Sianipar I. R. Matsui C. Minami N. Gan X. Deng L. Hotta H.. (2015). Physical and Functional Interaction Between Hepatitis C Virus NS5A Protein and Ovarian Tumor Protein Deubiquitinase 7B. Microbiol. Immunol. 59, 466–476. doi: 10.1111/1348-0421.12278 26112491

[B53] Singh V. Finke-Isami J. Hopper-Chidlaw A. C. Schwerk P. Thompson A. Tedin K. (2017). Salmonella Co-Opts Host Cell Chaperone-Mediated Autophagy for Intracellular Growth. J. Biol. Chem. 292, 1847–1864. doi: 10.1074/jbc.M116.759456 27932462 PMC5290957

[B54] Sooparb S. Price S. R. Shaoguang J. Franch H. A. (2004). Suppression of Chaperone-Mediated Autophagy in the Renal Cortex During Acute Diabetes Mellitus. Kidney Int. 65, 2135–2144. doi: 10.1111/j.1523-1755.2004.00639.x 15149326

[B55] Su W. C. Chao T. C. Huang Y. L. Weng S. C. Jeng K. S. Lai M. M. (2011). Rab5 and Class III Phosphoinositide 3-Kinase Vps34 are Involved in Hepatitis C Virus NS4B-Induced Autophagy. J. Virol. 85, 10561–10571. doi: 10.1128/JVI.00173-11 21835792 PMC3187495

[B56] Tekirdag K. Cuervo A. M. (2018). Chaperone-Mediated Autophagy and Endosomal Microautophagy: Joint by a Chaperone. J. Biol. Chem. 293, 5414–5424. doi: 10.1074/jbc.R117.818237 29247007 PMC5900761

[B57] Wang J. Kang R. Huang H. Xi X. Wang B. Wang J.. (2014). Hepatitis C Virus Core Protein Activates Autophagy Through EIF2AK3 and ATF6 UPR Pathway-Mediated MAP1LC3B and ATG12 Expression. Autophagy 10, 766–784. doi: 10.4161/auto.27954 24589849 PMC5119055

[B58] Wang Z. Li Y. Yang X. Zhao J. Cheng Y. Wang J. (2020). Mechanism and Complex Roles of HSC70 in Viral Infections. Front. Microbiol. 11, 1577. doi: 10.3389/fmicb.2020.01577 32849328 PMC7396710

[B59] Wang Y. Martinez-Vicente M. Krüger U. Kaushik S. Wong E. Mandelkow E. M.. (2009). Tau Fragmentation, Aggregation and Clearance: The Dual Role of Lysosomal Processing. Hum. Mol. Genet. 18, 4153–4170. doi: 10.1093/hmg/ddp367 19654187 PMC2758146

[B60] Wang X. Tanaka N. Hu X. Kimura T. Lu Y. Jia F.. (2019). A High-Cholesterol Diet Promotes Steatohepatitis and Liver Tumorigenesis in HCV Core Gene Transgenic Mice. Arch. Toxicol. 93, 1713–1725. doi: 10.1038/s41467-018-06931-6 31004178

[B61] Wing S. S. Chiang H. L. Goldberg A. L. Dice J. F. (1991). Proteins Containing Peptide Sequences Related to Lys-Phe-Glu-Arg-Gln are Selectively Depleted in Liver and Heart, But Not Skeletal Muscle, of Fasted Rats. Biochem. J. 275 (Pt 1), 165–169. doi: 10.1042/bj2750165 2018472 PMC1150027

[B62] Wong E. Cuervo A. M. (2010). Autophagy Gone Awry in Neurodegenerative Diseases. Nat. Neurosci. 13, 805–811. doi: 10.1038/nn.2575 20581817 PMC4038747

[B63] World Health Organization (2021). Global Progress Report on HIV, Viral Hepatitis and Sexually Transmitted Infections, 2021 (Geneva: World Health Organization).

[B64] Yang Y. Klionsky D. J. (2020). Autophagy and Disease: Unanswered Questions. Cell Death Differ. 27, 858–871. doi: 10.1038/s41418-019-0480-9 31900427 PMC7206137

